# Application of Artificial Intelligence in Anatomical Structure Recognition of Standard Section of Fetal Heart

**DOI:** 10.1155/2023/5650378

**Published:** 2023-01-24

**Authors:** Huiling Wu, Bingzheng Wu, Fangping Lai, Peizhong Liu, Guorong Lyu, Shaozheng He, Jiangfeng Dai

**Affiliations:** ^1^Department of Ultrasound, The Second Affiliated Hospital of Fujian Medical University, Quanzhou 362000, China; ^2^College of Engineering, Huaqiao University, Quanzhou 362021, China; ^3^Collaborative Innovation Center for Maternal and Infant Health Service Application Technology, Quanzhou Medical College, Quanzhou 362011, China

## Abstract

Congenital heart defect (CHD) refers to the overall structural abnormality of the heart or large blood vessels in the chest cavity. It is the most common type of fetal congenital defects. Prenatal diagnosis of congenital heart disease can improve the prognosis of the fetus to a certain extent. At present, prenatal diagnosis of CHD mainly uses 2D ultrasound to directly evaluate the development and function of fetal heart and main structures in the second trimester of pregnancy. Artificial recognition of fetal heart 2D ultrasound is a highly complex and tedious task, which requires a long period of prenatal training and practical experience. Compared with manual scanning, computer automatic identification and classification can significantly save time, ensure efficiency, and improve the accuracy of diagnosis. In this paper, an effective artificial intelligence recognition model is established by combining ultrasound images with artificial intelligence technology to assist ultrasound doctors in prenatal ultrasound fetal heart standard section recognition. The method data in this paper were obtained from the Second Affiliated Hospital of Fujian Medical University. The fetal apical four-chamber heart section, three vessel catheter section, three vessel trachea section, right ventricular outflow tract section, and left ventricular outflow tract section were collected at 20-24 weeks of gestation. 2687 image data were used for model establishment, and 673 image data were used for model validation. The experiment shows that the map value of this method in identifying different anatomical structures reaches 94.30%, the average accuracy rate reaches 94.60%, the average recall rate reaches 91.0%, and the average F1 coefficient reaches 93.40%. The experimental results show that this method can effectively identify the anatomical structures of different fetal heart sections and judge the standard sections according to these anatomical structures, which can provide an auxiliary diagnostic basis for ultrasound doctors to scan and lay a solid foundation for the diagnosis of congenital heart disease.

## 1. Introduction

Now, ultrasonic examination has been widely used in various medical diagnosis and treatment. It has the advantages of convenient operation, noninvasive, nonradiation, relatively low price, real-time observation, etc. Specifically in the prenatal examination of the fetus, ultrasound has become the preferred examination method [1]. It can not only observe the growth and development of the fetus but also conduct structural screening of the fetus. However, the standardization of the ultrasound section seriously affects the accuracy of the diagnosis of fetal structural screening. Therefore, it is extremely important to obtain the standard ultrasound section. Observation of the standard section is conducive to early detection of fetal structural abnormalities, especially fetal congenital cardiac structural abnormalities. Congenital heart defect (CHD) refers to the structural abnormality of the heart or large blood vessels in the thoracic cavity [2], which is the most common and easily missed congenital defect [3], and its incidence rate accounts for about 30% of congenital defects. With the development of science and economy, people's demand and desire for early screening and diagnosis of CHD are also growing. The diagnosis of CHD by fetal prenatal echocardiography is helpful to observe the fetal abnormalities and take reasonable measures in the follow-up treatment, so as to avoid the occurrence of hemodynamic damage [4]. At present, the best time for cardiac screening is the second trimester of pregnancy [5]. The four-chamber heart section (4CH), right ventricular outflow tract section (RVOT), left ventricular outflow tract section (LVOT), three vessel trachea section (3VT) and three vessel section (3VV) have five sections [6], and each section has cardiac anatomical structures and important adjacent structures that need attention. Ultrasound physicians conduct screening and diagnosis by identifying these sections and judging and analyzing the location and size of each atrium and ventricle, the connection relationship with corresponding arteries, and the corresponding location and size of major cardiac vessels.

However, ultrasound physicians to identify and determine the structure form and position if its normal that is more subjective evaluation method often need to spend a lot of time and energy but is also affected by many factors, such as fetal position, pregnant women amniotic fluid, different ultrasound model imaging differences, and different doctors of clinical knowledge level differences, which can lead to clinical diagnosis efficiency that is low. This study is aimed at establishing an effective auxiliary identification model to reduce the impact of negative factors on sonographers and improve the identification efficiency of the standard fetal heart ultrasound section anatomy, so as to help physicians improve the efficiency of fetal heart prenatal examination.

## 2. Related Work

The conventional prenatal fetal heart ultrasound screening method is to identify the current scanning section, preserve it, and use it later through the manual manipulation of the ultrasound instrument by the ultrasound doctor. Different ultrasound physicians may have different cognition and recognition methods for different sections, which lead to differences in the standard sections collected and make deviations in subsequent research and diagnosis. And the manual screening method requires doctors to spend a lot of time and energy in the prenatal cardiac ultrasound screening training and screening process. Therefore, the research can effectively improve the efficiency of prenatal fetal heart screening. The automatic identification and classification of the standard section of cardiac ultrasound and its anatomical structure are of great significance to prenatal ultrasound.

With the application and development of artificial intelligence (AI) in various fields, computers have also attracted considerable attention in medical research. As early as 2008, Liu et al. [7] used template matching to automatically search the best cross-sections of three-dimensional echocardiography and proved that AI is superior to manual search in searching these cross-sections. Yi and Babyn [8] improved the efficiency of AI in recognizing CT images by adjusting relevant parameters. In 2015, Yuhuan et al. [9] designed a navigation visualization system for standard transesophageal echocardiography, which realized the real-time navigation function of transesophageal probe, stereoscopically presented the collection technology operation of transesophageal echocardiography, effectively helped clinicians to enhance their understanding of the three-dimensional structure of the heart, master the collection technology of echocardiography, and accurately analyze and diagnose cases. In 2018, Madani et al. [10] used the deep convolution generation countermeasure network (DCGAN) to synthesize chest X-ray images, and used two GANs to generate normal and abnormal chest X-ray images, and evaluated the image quality of the results from classification and radiologist grading. In the same year, Zhang et al. [11] used GAN for data enhancement to improve the accuracy of tissue images. AI has also been applied to prenatal ultrasound examination of fetal face and brain. Chen [12] used deep learning (DL) to automatically measure the width of fetal lateral ventricle in 2D ultrasound images. Xie et al. [13] trained the algorithm in three aspects, fetal skull image segmentation, image classification, and focus location, and verified that DL can train to segment and classify normal and abnormal images. It can also provide the image of lesion location. Lei et al. [14] used Fisher vector to automatically recognize fetal facial structure and achieved good results, indicating that AI can effectively recognize fetal facial structure and is expected to be applied to other fetal organs. However, the application of machine learning (ML) and DL in the fetal heart is still in its infancy. In some studies [15], by collecting the echocardiographic video records of normal controls, patients with ventricular septal defect (VSD) and patients with atrial septal defect (ASD), DL was used to establish the research model and achieve the diagnostic effect with an accuracy rate of more than 90%. Xu et al. [16] conducted a large number of experiments on the four-chamber view dataset, which showed that DW-Net had a good segmentation effect on the four-chamber view. The crossapplication between AI and medicine is realized, which shows that AI is feasible and has a good application prospect in the heart.

The structure of each standard section of 2D ultrasound image of the fetal heart is similar. The age of pregnant women and the thickness of abdominal wall fat layer, the gestational age of the fetus, fetal orientation, different ultrasound examination instruments, and different ultrasound physicians will affect the imaging quality. Therefore, in medical image processing, recognition, and classification, ML based on traditional manual features and DL based on depth features are mainly adopted at present. Traditional manual classification is a semisupervised learning method, which requires manual preprocessing of the input image and screening of image candidate regions. Then, the feature extraction algorithm is used to extract image features, and finally, the classification algorithm is used to classify the extracted features. Although it has made some achievements, it also exposes its inherent disadvantages. The method of region selection by sliding window makes time consuming and window redundant. The instability of image quality changes leads to poor robustness and generalization of manual classification methods. Moreover, the complex algorithm steps also make detection efficiency slow and accuracy difficult to achieve satisfactory results. The traditional manual classification methods have been difficult to meet the needs of high performance image target detection. DL, a fully automatic learning mode, has aroused people's interest and attention. Automatic recognition and classification technology based on DL has gradually been introduced into automatic recognition and classification tasks in the field of medical images. DL is a network structure with multiple hidden layers and multiple perceptrons, which can describe image features in a deeper and higher dimension. It reduces the process of manual feature extraction and avoids the errors caused by this part [17]. The application of DL in image recognition is mainly divided into deep belief network (DBN), convolution neural network (CNN) [18], recurrent neural network (RNN), and generic adversary network (GAN). It has been proved in previous studies that CNN can effectively process various image tasks [19, 20]. In this study, YOLOV5 in CNN is selected to build an experimental model. YOLOV5 is a single-stage method, which directly obtains the final detection results and conducts intensive sampling at different locations of the image. During sampling, different aspect ratios can be used, and then CNN can be used to extract features. The greatest advantage is high speed [21].

Most of the scholars in the above articles have made good progress in their respective research fields, providing a solid foundation for the development of medical AI and smart medicine. However, the above research also has more or less problems. First, the applicability of the research fields is low, and it is difficult to expand to other ultrasonic images. Second, some methods are no longer applicable to the current ultrasonic image and AI progress and cannot be comparable to the new methods in terms of evaluation indicators.

In view of the above problems, this study is aimed at using AI and ultrasound technology to find a more convenient and efficient way to classify and identify the basic sections of the fetal heart, so as to reduce the differences in recognition and collection of the basic sections of the fetus brought by different doctors, different instruments, and different regions, and AI can partially replace and promote the long-term professional training of grassroots doctors. It is helpful for ultrasound doctors to control the quality of ultrasound images in their daily work, improve the primary diagnosis rate of CHD in China, and lay a foundation for early diagnosis and timely treatment of children's congenital heart disease.

The main contribution of this paper is to collect the standard sections of 2D ultrasound fetal heart in stages, and it is expected to establish a large fetal heart ultrasound database in China. At the same time, this paper proposes a fetal heart standard recognition model (U-Y-net) based on YOLOV5 depth convolution neural network. On the basis of repeated tests, through the comparison between different models, the comparison between data of different models, and the comparison between multiple doctors with different qualifications, it is proved that this method can effectively identify the fetal heart ultrasonic standard section and has the potential to assist ultrasound doctors in screening the fetal heart standard section.

## 3. Materials and Methods

### 3.1. Data Source and Data Collection

**Figure 1:**
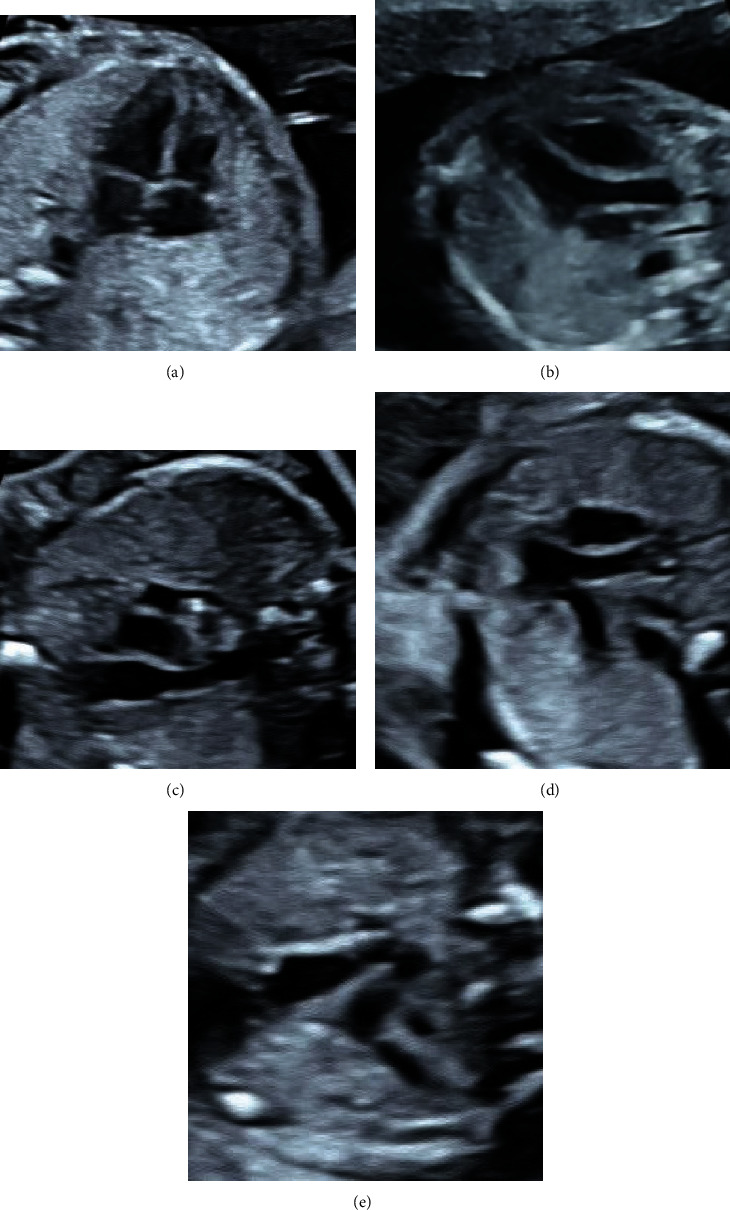
Schematic diagram of standard five section of the heart. (a) Four-chamber heart section. (b) Section of left ventricular outflow tract section. (c) Right ventricular outflow tract section. (d) Three vessel section. (e) Three vessel trachea section.

The five standard sectional images of the fetal heart were collected by doctors in charge of and above the Ultrasound Department of the Second Affiliated Hospital of Fujian Medical University according to the standard sectional images (Figure 1) defined in the ISUOG [6] guidelines. The identification of the standard sectional images after collection is based on expert judgment. From January 2020 to June 2022, 1300 pregnant women with an average age of 33 ± 2 years who underwent fetal echocardiography during their mid pregnancy (18-24^+6^ weeks of gestation) in the Second Affiliated Hospital of Fujian Medical University were selected. This study was approved by the Medical Ethics Committee of our hospital, and all patients signed an informed consent form. Finally, a total of 3360 pieces of fetal heart ultrasonic five standard sections data were added to the experiment, which were divided into training set and verification set. In addition, 482 pieces of fetal heart ultrasonic five sections including nonstandard sections were collected as the experiment set (image sizes include 704^∗^561, 720^∗^576, 720^∗^370, 768^∗^576, 1260^∗^910) (Table 1).

Inclusive criteria were as follows: ① the gestational age of ultrasound was consistent with the actual gestational age; ② the image is clear, the target structure is located in the middle of the image and occupies more than 1/2 of the whole image, and the background is pure without artifacts; ③ there are no measuring points, lines, and color Doppler blood flow signals in the image; and ④ postpartum confirmed that the fetus had no heart malformation or other structural malformations.

Exclusion criteria were as follows: ① unsatisfactory display of cardiac structure due to obesity of pregnant women, image jitter, and other factors; ② the structure of the image heart is incomplete; and ③ ultrasonography or postpartum confirmed fetal abnormalities.

Image acquisition instruments mainly include DC-8, Resona 8 (Mindray, China), Voluson E10, Voluson E8, Voluson E6, Rietta 70, HI Vision Preiru, Erlangshen (Hitachi, Japan), Logiq P6, Expert 730 (General Electric, USA), Aixlorer (Acoustics, France), and Sequoia 512 (Siemens, Germany). The 5.0 MHz convex array probe equipped with the above 12 kinds of color Doppler ultrasound diagnostic instruments was used to scan the fetal heart rate during the middle pregnancy.

### 3.2. Model Architecture

**Figure 2:**
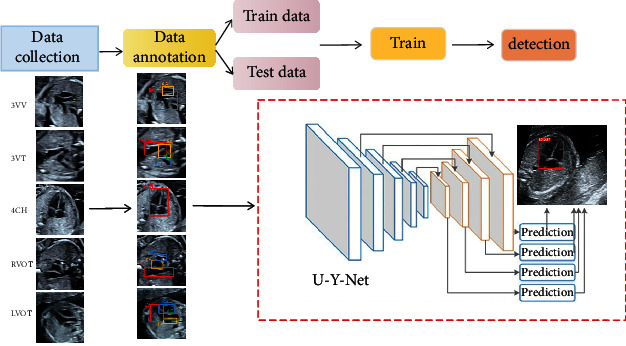
Flow chart of model construction: model construction and model verification after image preprocessing.

In the standard section scanning stage of real-time fetal heart ultrasound, there are a series of problems, such as the uneven level of doctors and different imaging effects of scanning equipment. As a result, the obtained sections do not meet the requirements of the code standards or even do not meet the standards, resulting in a higher misdiagnosis rate at the later stage of clinical diagnosis of congenital heart defects. In this paper, according to the ultrasound code, a more perfect definition standard of fetal heart ultrasound standard section is established, and on this basis, a high-performance AI recognition model (U-Y-net) of YOLOV5 [22–24] fetal heart standard section based on SIoU module is proposed. The goal of accurately identifying standard sections and training ultrasound physicians is not only solved in the real-time scanning phase. It also provides a more unified standard for the clinical diagnosis stage and reduces the incidence of misdiagnosis. The specific experimental flow chart is shown in Figure 2.

#### 3.2.1. Data Preprocessing Process

4CH is the most basic section to evaluate the fetal heart, and it is also the relatively most accessible section, which can visually see the left atrium (LA), right atrium (RA), left ventricle (LV), and right ventricle (RV). The left and right atria are roughly equal in size to the left and right ventricles, with the mitral and tricuspid valves and (or) the ventricular septum, the atrial septum, and the foramen ovale visible. The thickness of the ventricular wall should be approximately equal [25]. The majority of congenital heart defects including single ventricular system, LV dysplasia, atrial septal defect, and downward diaplacement of the malformed tricuspid value were judged by observing the structure of 4CH.

However, it is difficult to see isolated outflow tract lesions only by relying on 4CH. We can jointly judge RV, ascending aorta (AA), superior vena cava (SVC), LA, and LV in RVOT. The structure and location of the aorta (aorta, AO) and RV assist in screening outflow tract diseases, such as irregular cone arrangement, ventricular septal defect, abnormal semilunar valve, coarctation of the aorta, tetralogy of Fallot, and transposition of large vessels [26], but the observation and acquisition of the outflow tract section not only depend on the operation level of the ultrasound doctor but also depend on factors such as fetal position. When it is difficult to obtain the outflow channel section, 3VV and 3VT provide another method to evaluate the outflow channel anomaly, which is technically easier. 3VV includes pulmonary artery (PA) and AA, in which PA has a “Y” bifurcation; 3VT includes AO, main pulmonary artery (MPA), and SVC cross-section, with trachea (T) behind SVC [27]. The difference between RVOT and 3VT is mainly the aortic arch and T, which is helpful to better identify catheter dependent lesions. 3VT uses T as the reference point to help identify and compare blood vessels.

**Figure 3:**
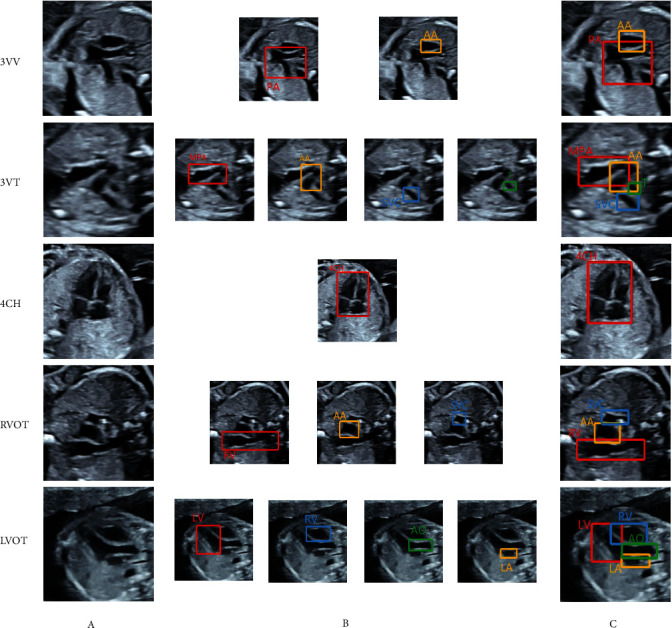
Schematic diagram of standard section and structure annotation. (a) Ultrasonic standard five section. Note of each anatomical structure in section (b). (c) Complete annotation drawing of each section.

4CH, LVOT, RVOT, 3VV, and 3VT are currently the most commonly used and most basic sections of prenatal fetal heart screening, which can complement each other and observe the fetal heart in many aspects. Therefore, this study selected these five standard sections as the research object, and the standard structure is shown in Table 2 and Figure 3.

#### 3.2.2. Fetal Heart Recognition Network

**Figure 4:**
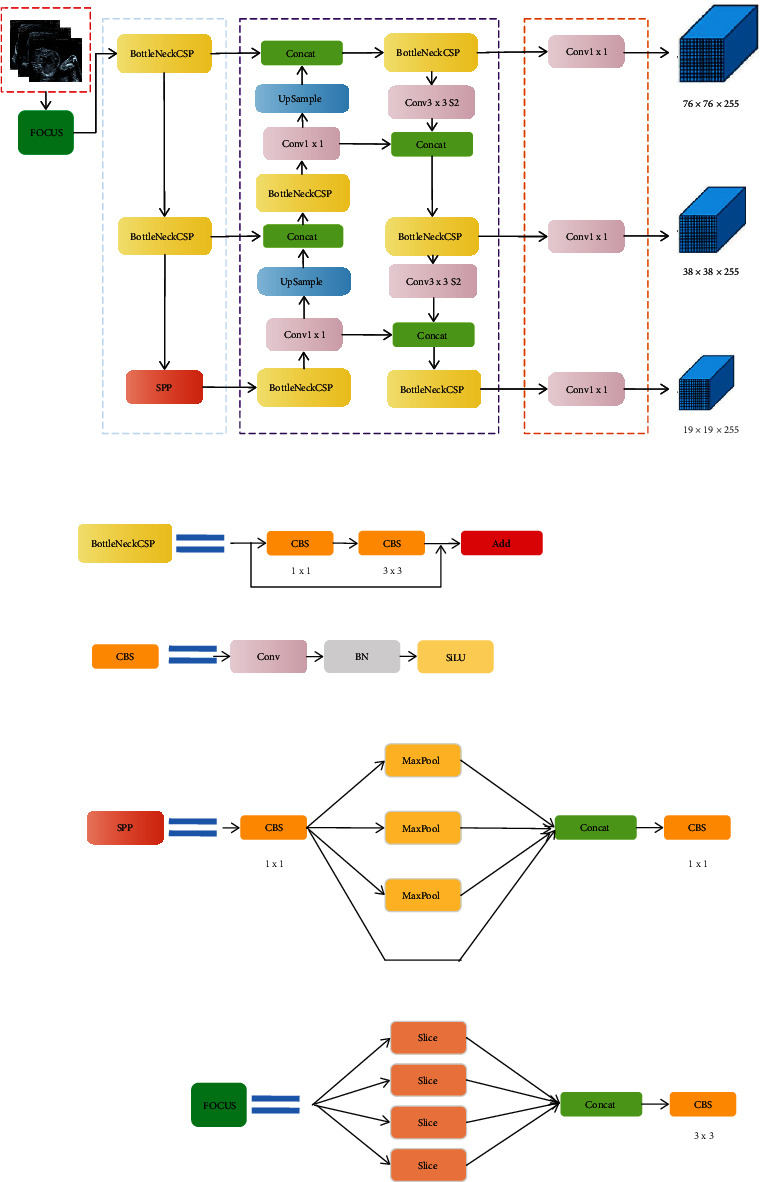
Fetal heart recognition network.

*(1) Network Architecture*. The fetal heart recognition network in this paper is based on YOLOV5, and the detailed structure is shown in Figure 4. It is mainly composed of Input, Backbone, Neck, and Prediction forms. The Input section is used for image input and data preprocessing. Backbone is responsible for the aggregation of different images and the formation of image features. Neck contains a series of mixed and combined image features and passes them to Prediction. Prediction predicts image features and generates a prediction bounding box.

The backbone of fetal heart recognition network is CSPDarknet. It is mainly composed of Focus, convolutional batch normalization SiLU (CBS), CSP1_X, CSP2_10, and spatial pyramid pooling (SPP) composition. We can see from Figure 3 that the first layer of the trunk is Focus, which stacks four adjacent positions of the image through slice to reduce the loss of original information and speed up the calculation. CBS blocks are formed into convolution layers, followed by batch normalization (BN) layers and SiLU activation functions.

The network mainly applies two CSP structures, namely, CSP1_X and CSP2_X. CSP1_X means the module contains *x* bottlenecks, and CSP2_X indicates that the second CBS in the first line of the module contains 2 ^∗^ X bottlenecks. The network integrates gradient changes into the feature map through two CSP structures, which solves the problem of gradient information duplication and gradient disappearance in other networks, and effectively reduces network parameters while ensuring accuracy and speed. The SPP module is mainly composed of CBS blocks and the largest pool layer. The input terminals successively pass through the CBS block and the three 3 × 3, 5 × 5, and 7 × 7's core parallel max pool and then concat fusion, effectively increasing the size range of image features.

Based on BN layers, it is used to normalize the feature maps after the convolutional layer to accelerate the network learning, which also has a certain regularization effect. During training, BN needs to learn the mean and variance of a minibatch data and then use this information to normalize. In the reasoning process, in order to accelerate, BN will be integrated into its upper convolution, and the two-step operation is turned into a step to achieve the purpose of acceleration. YOLOV5 what is realized is a small content of the parameter restructure, which is to merge the convolution and BN layers into a new convolution, but contains the characteristics of the BN layer.

SiLU activation function in YOLOV5 essentially adds to the ReLU activation function unbound and smooth and nonmonotonic to the sigmoid activation function, so SiLU is an improved version of Sigmoid and ReLU. The SiLU performs better than the ReLU on the deep model.

In this paper, the feature pyramid network (FPN) plus path aggregation network (PAN) structure is adopted in the Neck layer of fetal heart recognition network. FPN structure transfers and fuses advanced feature information through upsampling, while PAN structure transfers strong positioning features from the bottom to the top. We combine these two structures to aggregate features on different detection layers and then generate feature maps of different scales to detect targets on specific scales. Finally, region proxies are generated on four different scales, whose sizes are 1/8, 1/16, 1/32, and 1/64 of the height and width of the original input image, and the corresponding feature maps are combined to effectively detect structures of different sizes.

*(2) Loss Function*. In the training process, excellent loss function has a crucial impact on the results. This model will number is defined as three parts, and the formula is as follows:

(1) $$\begin{array}{c} L=a^{*}L_{\text{CLS}}+b^{*}L_{\text{OBJ}}+c^{*}L_{\text{BOX}}. \end{array}$$

*L*_CLS_ represents the category loss function, indicating whether there is a target of type *j* in prediction box *i*. If there is a target of type j, it is equal to 1; otherwise, it is equal to 0. *x*_*ij*_ is the predicted output value, and *N*_pos_ is the number of positive samples. The formula is as follows:

(2) $$\begin{array}{c} L_{\text{CLS}}=-\frac{1}{N_{\text{pos}}}\left(y_{ij}\log\left(\text{sigmoid}\left(x_{ij}\right)\right)+\left(1-y_{ij}\right)\log\left(1-\text{sigmoid}\left(x_{ij}\right)\right)\right), \end{array}$$

(3) $$\begin{array}{c} \text{sigmoid}\left(x_{ij}\right)=\frac{e^{x_{ij}}}{\sum_{n=1}^{N}e^{x_{n}}}. \end{array}$$

The sigmoid(*x*_*ij*_) in the classification loss function is shown as above:

*L*_OBJ_ represents the target confidence loss function. The sample pair obtained from positive sample matching is calculated. One is the target confidence score in the prediction box. The other is the IoU value of the prediction box and the corresponding target box, which is used as the ground truth. *L*_OBJ_ indicates the degree of overlap between the prediction box and the GT box. *x*_*i*_ is the predicted value of the network for the current target, and *N* is the sum of positive and negative samples. Both calculate the binary cross entropy to obtain the final target confidence loss. The formula is as follows:

(4) $$\begin{array}{c} L_{\text{OBJ}}=-\frac{1}{N}\left(y_{\text{i}}\log\left(\text{sigmoid}\left(x_{i}\right)\right)+\left(1-y_{i}\right)\log\left(1-\text{sigmoid}\left(x_{i}\right)\right)\right), \end{array}$$

(5) $$\begin{array}{c} \text{sigmoid}\left(x_{i}\right)=\frac{1}{1+e^{-x_{i}}}. \end{array}$$

The sigmoid(*x*_*i*_) in the classification loss function is shown as above:

*L*_BOX_ represents the bounding box regression loss function. In the original YOLOV5 model, CIoU loss function is used. Some scholars have proposed a new loss function, SIoU. SIoU introduces the vector angle between the real box and the prediction box and redefines the related loss function. In this paper, SIoU is used as *L*_BOX_.

The SIoU loss function consists of the following four cost functions:

1. Angle cost

**Figure 5:**
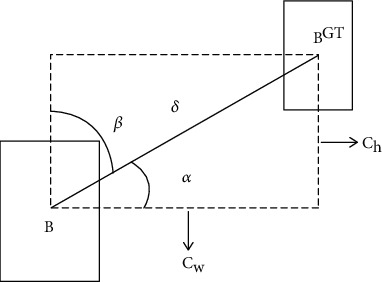
Curve of angle cost.

The curve of angle cost is shown in Figure 5 and is defined as follows.

(6) $$\begin{array}{c} \Lambda =1-2^{*}\sin^{2}\left(\arcsin\left(\frac{c_{h}}{\sigma }\right)-\frac{\pi }{4}\right)=\cos\left(2^{*}\left(\arcsin\right)\left(\frac{c_{h}}{\sigma }\right)-\frac{\pi }{4}\right), \end{array}$$

where *c*_*h*_ is the height difference between the center points of the real box and the prediction box, and *σ* is the distance between the center points of the real box and the prediction box. In fact, arcsin(*c*_*h*_/*σ*) is equal to the angle *α*.

(7) $$\begin{array}{c} \frac{c_{h}}{\sigma }=\sin\left(\alpha \right), \end{array}$$

(8) $$\begin{array}{c} \sigma =\sqrt{{\left(b_{c_{x}}^{gt}-b_{c_{x}}\right)}^{2}-{\left(b_{c_{y}}^{gt}-b_{c_{y}}\right)}^{2}}, \end{array}$$

(9) $$\begin{array}{c} c_{h}=\max\left(b_{c_{y}}^{gt},b_{c_{y}}\right)-\min\left(b_{c_{y}}^{gt}-b_{c_{y}}\right). \end{array}$$

*b*_*c*_*y*__^*gt*^ − *b*_*c*_*y*__ is the center coordinate of the real box, and *b*_*c*_*x*__^*gt*^ − *b*_*c*_*x*__ is the center coordinate of the prediction box. It can be noted that when *α* is *π*/2 or 0, the angle loss is 0. If *α* < *π*/4 in the training process, it is minimized to *α*; otherwise, it is *β*.

(2) Distance cost

**Figure 6:**
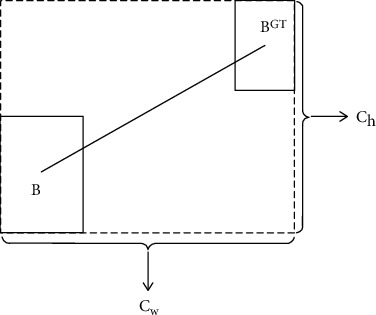
Curve of distance cost.

The curve of distance cost is shown in Figure 6 and is defined as follows:

(10) $$\begin{array}{c} \Delta =\underset{t=w,h}{\sum }\left(1-e^{-\gamma pt}\right)=2-e^{-\gamma px}-e^{-\gamma py}, \end{array}$$

including

(11) $$\begin{array}{c} p_{x}={\left(\frac{b_{c_{x}}^{gt}-b_{c_{x}}}{c_{w}}\right)}^{2},p_{y}={\left(\frac{b_{c_{y}}^{gt}-b_{c_{y}}}{c_{h}}\right)}^{2},\gamma =2-\Delta . \end{array}$$

In the above, *c*_*w*_ and *c*_*h*_ are the width and height of the minimum bounding rectangle of the real box and the prediction box, respectively.

(3) Shape cost

Shape cost is defined as follows:

(12) $$\begin{array}{c} \Omega =\underset{t=w,h}{\sum }{\left(1-e^{-wt}\right)}^{\theta }={\left(1-e^{-w_{w}}\right)}^{\theta }+{\left(1-e^{-w_{t}}\right)}^{\theta }, \end{array}$$

including

(13) $$\begin{array}{c} w_{w}=\frac{\left|w-w^{gt}\right|}{\max\left(w,w^{gt}\right)},w_{h}=\frac{\left|h-h^{gt}\right|}{\max\left(h,h^{gt}\right)}. \end{array}$$

(*w*, *h*) and (*w*^*gt*^, *h*^gt^) are the width and height of the prediction box and the real box, to control the degree of attention to the shape loss. In order to avoid paying too much attention to the shape loss and reduce the movement of the prediction box, this stage uses genetic algorithm to calculate *θ*'s value close to 4, so the *θ* parameter range is [2, 6].

(4) IoU cost

**Figure 7:**
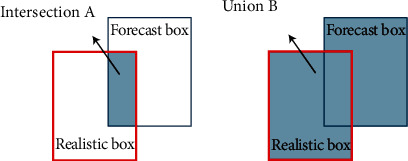
IoU loss.

See Figure 7 below for details: the ratio between the overlapping area of two regions and the union of the area (or the largest area, the smallest area):

To sum up the four cost functions, the final SIoU loss function is defined as follows:

(14) $$\begin{array}{c} L_{\text{BOX}}=1-IoU+\frac{\Delta +\Omega }{2}. \end{array}$$

## 4. Experiment and Analysis

### 4.1. Experimental Environment

The computer configuration of this experiment is as follows: Intel (*R*) Core (TM) i7-10700K is used as CPU, NVIDIA GeForce GTX-1080Ti is used as GPU, the video memory is 16 G, the memory is 32 G, the computer operating system is 64 bit Windows10, the programming software is PyCharm 2020.2.2 ×64, and the programming language is Python 3.7.

### 4.2. Evaluation Index

The verification set is used to verify the recognition effect of U-Y-net and the performance comparison between U-Y-net and different AI models. For the target detection model used, MAP, accuracy rate, and recall rate are important evaluation indicators for testing the algorithm model. However, in some scenarios, the calculation methods of recall rate and accuracy rate often have some contradictions. Here, we introduce the commonly used comprehensive evaluation index *F*-measure, which is the weighted harmonic average of the accuracy rate and recall rate. The higher the *F* value, the more effective the model is. The corresponding calculation formula is given below for the above evaluation indicators:

(15) $$\begin{array}{c} \text{Precision}=\frac{\text{TP}}{\text{TP}+\text{FP}}, \end{array}$$

(16) $$\begin{array}{c} \text{Recall}=\frac{\text{TP}}{\text{TP}+\text{FN}}, \end{array}$$

(17) $$\begin{array}{c} F1=\frac{2\times \text{Precision}\times \text{Recall}}{\left(\text{Precision}+\text{Recall}\right)}, \end{array}$$

(18) $$\begin{array}{c} \text{mAP}=\frac{\sum_{i=1}^{k}{\text{AP}}_{i}}{k}. \end{array}$$

True positive (TP) refers to the number of correct classifications after testing, false positive (FP) refers to the number of noncategory people who are wrongly classified after testing, true negative (TN) refers to the number of people who are not in this category correctly classified after the test, and false negative (FN) refers to the number of people who are wrongly classified after testing.

It can be seen from the above formula that the accuracy rate is equivalent to the positive predictive value, and the recall rate is equivalent to the sensitivity. The positive predictive value (PPV) will replace the accuracy rate, and the sensitivity (S) will replace the recall rate.

The experimental set is used to compare the identification efficiency of U-Y-net and doctors of different grades, calculate the sensitivity, specificity, positive predictive value, and accuracy of U-Y-net, junior doctors, and intermediate doctors for each section, and use the McNemar chi-square test for comparison between groups. *P* < 0.05 is considered statistically significant.

### 4.3. Test Results

#### 4.3.1. Recognition Results of U-Y-Net Model for Each Anatomical Structure

The overall recognition map value of U-Y-net on the standard section of fetal heart reached 94.3%, and the overall F1 score was 0.9277. The average of PPV was 94.60%, the average of S was 91.0%, the F1 score was 0.82~0.99, and the specificity was 99.2%~93.7%. The best effect is 4CH, the map reached 99.5%, PPV was 99.5%, S was 100%, the specificity was 99.2%, and the F1 score reached 0.9975. The LV's map reached 99.5%, PPV was 99.2%, S was 100%, the specificity was 98.4%, and the F1 score reached 0.9959. The worst effect was the trachea structure. The map was 86.9%, PPV was 90.9%, S was 75.5%, the specificity was 97.2%, and the F1 score was only 0.8249 (Table 3). From the analysis of the results, the recognition effect of all anatomical structures is more than 85%.

#### 4.3.2. Model Comparison Experiment

As U-Y-net is improved based on YOLOV5, it is compared with the original YOLOV5 and the commonly used deep learning model. The experiment is conducted in the same environment and the same data set.  Table 4 shows the model in this paper, and Fast-RCNN&Mobilenetv2, Fast-RCNN&Res-Net50, RetinaNet&Res-Net50, SSD&Res-Net50, VGG-Net&Res-Net50, and other tow stage networks are compared with the one-stage YOLOV5 model.

The U-Y-net has achieved 94.3% effect on the map, compared with the deep learning of the tow stage Fast-RCNN&Mobilenetv2, Fast-RCNN&Res-Net50, and RetinaNet&Res-Net50. With SSD&Res-Net50, VGG-Net&Res-Net50, and other methods, various evaluation indicators of U-Y-net are significantly ahead of each model, and the best Fast-RCNN&Mobilenetv2 can only reach 81.2% on the map. The map value of U-Y-net is 0.9% higher than YOLOV5.

#### 4.3.3. Comparative Experiment between Different Doctors and U-Y-Net

The sensitivity of U-Y-net to identify 4CH, 3VV, 3VT, and LVOT is higher than that of primary physicians, and the accuracy of U-Y-net to identify 3VV, RVOT, and LVOT is higher than that of primary physicians. The accuracy and sensitivity of U-Y-net in the identification of the overall section are higher than those of the primary level and are equivalent to those of the intermediate level (Table 5).

### 4.4. Discussion and Later Work

Ultrasonography is an important means of prenatal examination, through which most fetal malformations can be observed and diagnosed, especially for congenital heart defects. It is the most critical step for prenatal heart screening to identify and obtain the standard fetal heart ultrasound section. However, in the clinical screening work, the fetal heart structure is complex, the volume is small, and there are many adjacent organs. The recognition and acquisition of the standard section of the heart are easily affected by factors such as fetal position. Moreover, different doctors have different qualifications, different hospitals have different equipment, and different cognitions of the standard view of the heart lead to different time spent in scanning to obtain the standard view. These will lead to long inspection time, different image quality, and limited recognition accuracy. Therefore, this paper proposes a new classification method that can identify the standard section of fetal heart faster and more accurately. It is hoped that this method can be used in clinical practice in the future, to help doctors identify and obtain the standard section of heart, to help doctors train in prenatal echocardiography, and to improve the quality of the standard section of heart images.

U-Y-net has better performance than the commonly used deep learning model, but compared with YOLOV5, U-Y-net has a slight lead of 0.9% on the map and a slight decline in other evaluation indicators. The main reason for analysis is that the network model of tow stage has a better recognition effect on large targets. For such data as fetal heart ultrasonic anatomical structure, the fetal heart has similar standard sections, which are in the same round heart, and different points are in each small target. The YOLOV5 model can identify small targets well and can interact with the surrounding information for small targets. Therefore, the YOLOV5 model is significantly ahead of the network model of tow stage in the task of anatomical structure recognition of fetal heart standard section. Secondly, with the above-mentioned angle information, in the process of ultrasonic fetal heart standard section scanning, due to the different scanning methods of ultrasonic doctors, the anatomical structures of different sections scanned are different. The SIoU loss function used in this paper can pay more attention to angle information, so U-Y-net is better than the original YOLOV5.

The experiment in this paper proves that U-Y-net can effectively identify several important structures of the heart and has a good effect on the classification of five sections of the cardiac ultrasound standard, which is not only better than the original YOLOV5 but also better than the current commonly used deep learning model. In comparison with doctors with different qualifications, we find that U-Y-net has the same recognition efficiency as intermediate doctors. However, there are still some deficiencies in this paper, which will also be the goal of later research: the method of this paper refined the annotation of the structure and will consume more physician resources. This paper adopts the YOLOV5 method. Deep learning is limited by many aspects, such as data quantity, excellence of model, training time and resource consumption, and how to reasonably allocate all aspects of balance. This is also one of our later research directions. The recognition effect of trachea in many structures is relatively poor. How to better improve the recognition efficiency, increase the corresponding measurement function, and calculate the position relationship of each structure will be the focus of later research, providing more information for congenital heart defects and assisting ultrasound doctors in diagnosis. Later, we will continue to collect data, train models, and expand the database of fetal echocardiography standard sections.

## 5. Conclusion

CHD is the most common and easily missed congenital defect. With the development of society and the opening of the second child policy, people have a growing demand for early diagnosis of CHD. The key to diagnosing CHD is to identify and obtain standard sections and observe the anatomical relationship among them. In this paper, a new method of label recognition is proposed. By specifically identifying the local anatomical structure of a single standard section, the standard section of fetal heart can be more accurately located under the ISUOG standard, providing a new idea for the automatic standardization of prenatal fetal ultrasound examination procedures and creating favorable conditions for ultrasound physicians to learn the standard section of cardiac ultrasound. At the same time, this paper has established a depth convolution neural network model (U-Y-net) based on YOLOV5. Through experiments, it has been proved that U-Y-net can effectively recognize and classify many important structures of fetal heart and the standard five sections of fetal heart. In contrast experiments, U-Y-net is obviously superior to the commonly used depth learning model. U-Y-net's recognition effect on the five sections of the heart is better than that of primary physicians and comparable to that of intermediate physicians. At this stage, it can help doctors to reduce the adverse effects of prenatal screening of the fetal heart more quickly and accurately, laying a foundation for the automatic standardization of prenatal fetal ultrasound procedures, which is worthy of further exploration. U-Y-net has the function of assisting doctors to identify the sections of classification standards, helping doctors to identify important anatomical structures of the heart, and helping doctors to screen the potential value of CHD.

## Figures and Tables

**Table 1:** Data set allocation results.

Section type	Total data	Training set	Test set	Experimental set
3VV	439	296	62	81
3VT	860	601	176	83
4CH	1368	972	252	144
RVOT	593	407	89	92
LVOT	587	411	94	82
Total	3840	2687	673	482

**Table 2:** Display content and marking structure of standard section.

Standard section	Show contents	Dimension structure
4CH	Tricuspid valve attachment point, atrial septal endocardial pad and interventricular septum (crisscross), and left and right cardiac chambers are roughly the same size	Approximately symmetrical 4CH of atrium and ventricle
LVOT	RV, LV, LA, AO	LA, LV, RV, and AO
RVOT	RV, AA, and SVC connected with PA form a three vessel side-by-side image	RV, AA, SVC
3VV	MPA sends out left and right PA at the bifurcation, forming a “Y”-shaped structure and/or showing the ascending aorta, superior vena cava, and descending aorta	Bifurcated PA and AO
3VT	AO and MPA form a “V” structure, showing SVC and T	AO, MPA, SVC, and T

**Table 3:** Recognition results of each anatomical node by U-Y-net.

Anatomic structure	Map (%)	PPV (%)	S (%)	F1 score	Specificity (%)
4CH	99.5	99.5	100	0.9975	99.2
LV	99.5	99.2	100	0.9959	98.4
LA	97.9	95.0	92.0	0.9348	98.4
AO	90.3	90.0	87.0	0.8847	93.7
RV	89.4	92.2	84.1	0.8796	96.5
AA	94.2	93.7	89.2	0.9139	97.4
SVC	95.1	99.2	88.7	0.9366	97.9
PA	94.8	93.9	96.4	0.9513	99.6
MPA	95.7	92.5	96.8	0.9460	97.2
T	86.9	90.9	75.5	0.8249	97.2
All	94.3	94.6	91.0	0.9277	

**Table 4:** Comparison experiment of different models.

Model	Map (%)	PPV (%)	S (%)	F1 score
Fast-RCNN&Mobilenetv2	81.20	84.20	82.20	0.8318
Fast-RCNN&Res-Net50	75.60	77.60	80.20	0.7888
RetinaNet&Res-Net50	77.40	81.40	83.80	0.8258
SSD&Res-Net50	66.20	60.40	72.20	0.6577
VGG-Net&Res-Net50	79.60	74.20	86.40	0.7983
YOLOV5	93.40	94.90	92.70	0.9379
U-Y-net	94.30	94.60	91.00	0.9277

**Table 5:** Comparative experimental results between different doctors and U-Y-net.

Section	Method	S (%)	Specificity (%)	PPV (%)	Accuracy (%)
4CH	Junior physician	90.28	38.89	83.87	80.00
	Intermediate physician	95.83^a^	55.56	89.61	87.78
	U-Y-net	98.6^a^	33.33	85.45	85.00

3VV	Junior physician	74.07	20.00	88.24	68.13
	Intermediate physician	86.41^a^	40.00	92.11	81.32^a^
	U-Y-net	91.36^a^	40.00	92.50	85.71^a^

3VT	Junior physician	74.07	19.35	73.40	65.79
	Intermediate physician	96.39^a^	16.67	80.00	73.68
	U-Y-net	97.53^a^	25.81	77.45	76.32

RVOT	Junior physician	76.09	50.00	73.40	71.43
	Intermediate physician	82.61	70.00	92.68	80.36
	U-Y-net	88.04	80.00	95.29	86.61^a^

LVOT	Junior physician	66.27	52.38	84.62	64.08
	Intermediate physician	85.37^a^	71.43	92.11	82.52^a^
	U-Y-net	82.92^a^	76.19	93.15	81.55^a^

Whole	Junior physician	79.67	63.56	83.66	71.17
	Intermediate physician	90.04^a^	51.35	88.93	82.80^a^
	U-Y-net	87.72^a^	58.95	91.90	83.17^a^

Note: ^a^compared with the primary level, *P* < 0.05 representative is statistically significant.

## Data Availability

The five standard sectional images of the fetal heart were collected by doctors in charge of and above the Ultrasound Department of the Second Affiliated Hospital of Fujian Medical University according to the standard sectional images (Figure 1) defined in the ISUOG guidelines. The identification of the standard sectional images after collection is based on expert judgment. From January 2020 to June 2022, 1300 pregnant women with an average age of 33 ± 2 years who underwent fetal echocardiography during their mid pregnancy (18-24 weeks) in the Second Affiliated Hospital of Fujian Medical University were selected. This study was approved by the Medical Ethics Committee of our hospital, and all patients signed an informed consent form. Finally, a total of 3360 pieces of fetal heart ultrasonic five standard sections data were added to the experiment, which were divided into training set and verification set.
